# Precision measurement of radioactivity in gamma-rays spectrometry using two HPGe detectors (BEGe-6530 and GC0818-7600SL models) comparison techniques: Application to the soil measurement

**DOI:** 10.1016/j.mex.2016.12.003

**Published:** 2016-12-31

**Authors:** Guembou Shouop Cebastien Joel, Samafou Penabei, Moyo Maurice Ndontchueng, Gregoire Chene, Eric Jilbert Nguelem Mekontso, Alexandre Ngwa Ebongue, Motapon Ousmanou, Strivay David

**Affiliations:** a Atomic and Nuclear Spectroscopy, Archeometry, University of Liège, Bat. B15 Sart Tilman, 4000 Liege 1, Belgium; b Department of Physics, Faculty of Science, University of Douala, P.O. Box 24157, Douala, Cameroon; c Centre for Atomic Molecular Physics and Quantum Optics, University of Douala, P.O. Box 8580, Douala, Cameroon; d National Radiation Protection Agency, P.O. Box 33732, Yaounde, Cameroon

**Keywords:** HPGe, Gamma spectrometry, Specific activity, BEGE-6530, GC0818-7600SL, Relative uncertainty

## Abstract

To obtain high quality of results in gamma spectrometry, it is necessary to select the best HPGe detector for particular measurements, to calibrate energy and efficiency of gamma detector as accurate as possible. To achieve this aim, the convenient detector model and gamma source can be very useful. The purpose of this study was to evaluate the soil specific activity using two HPGe model (BEGe-6530 and GC0818-7600SL) by comparing the results of the two detectors and the technics used according to the detector type. The relative uncertainty activity concentration was calculated for ^226^Ra, ^232^Th and ^40^K. For broad energy germanium detector, BEGe-6530, the relative uncertainty concentration ranged from 2.85 to 3.09% with a mean of 2.99% for ^226^Ra, from 2.29 to 2.49% with a means of 2.36% for ^232^Th and from 3.47 to 22.37% with a mean of 12.52% for ^40^K. For GC0818-7600SL detector, it was ranged from 10.45 to 25.55% with a mean of 17.10% for ^226^Ra, from 2.54 to 3.56% with a means of 3.10% for ^232^Th and from 3.42 to 7.65% with a mean of 5.58% for ^40^K. The average report between GC0818-7600SL model and BEGe-6530 model was calculated and showed the mean value of 3.36. The main study was based on the following points:

• Determination of The relative uncertainty activity concentration of ^226^Ra, ^232^Th and ^40^K
• Determination of the relative uncertainty related to the radium equivalent activity to compare the performance of the two detection systems
• Proved that the activity concentration determination in gamma spectrometry depended on the energy range emitted by a radionuclide.

This study showed that the standard deviation measurement was less important to the result realized with BEGe-6530 HPGe model. Our findings were demonstrated that the results of the Broad Energy Germanium detector were more reliable.

## Graphical abstract

**Figure:**
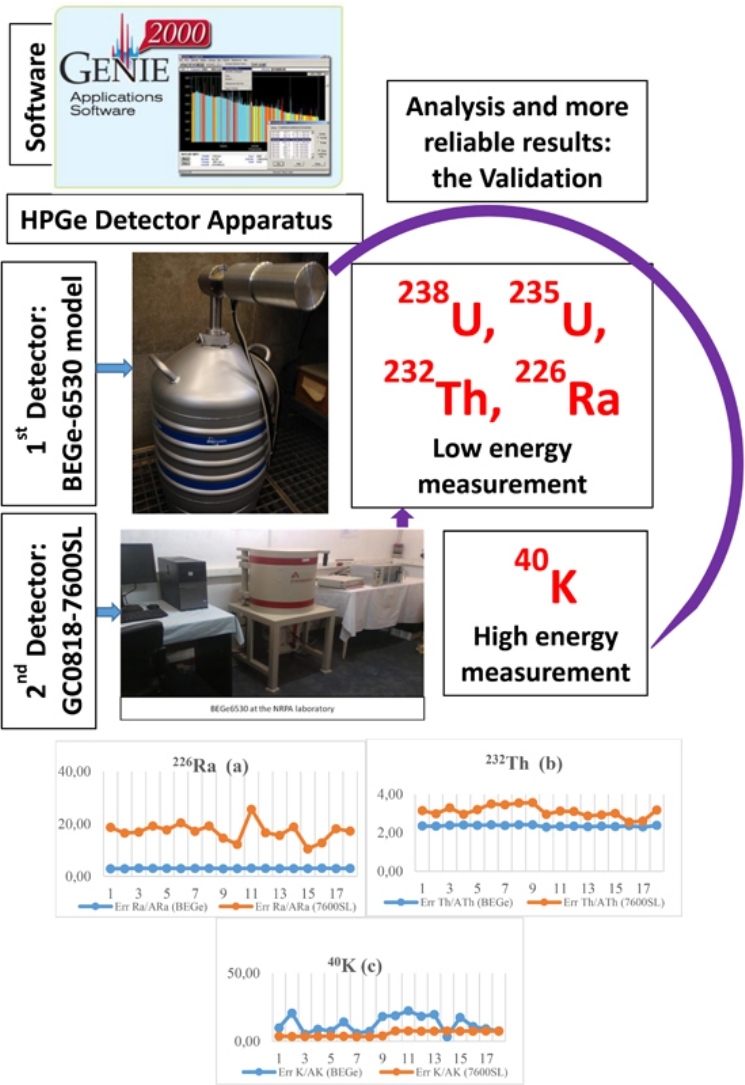

## Method details

Gamma-ray spectrometry is a non-destructive technics used to gauge electromagnetic radiation in the gamma-ray spectrum of radioactive sources. This is performed through the procedure of the counting and measuring the energy of individual photon emitted from different elements present in soil. The use of germanium detectors in high-resolution gamma-ray spectrometry is a standout amongst the most generally utilized strategies for the identification and quantification of unknown gamma-ray emitting radionuclides in sample [1], [2]. The estimation of gamma rays is valuable for the determination of the elemental sample composition of a wide assortment of sources. The measured energy of a gamma-ray corresponds to the type of element and its isotope, while the number of counts corresponds to the abundance of the radioactive source present in the measured sample with some little considerations. The process of measuring a gamma ray begins at the radioactive source, which emits high energy photons during its unstable radioactive decay [3]. This spectrometry technique requires earlier learning of the photo-peak efficiency of the detector in the counting geometry for each photon energy.

In any case, measures crevices with various equipment in gamma spectrometry are nowadays a real challenge for scientists: to locate the most efficiency and enhance the measurement time in the lab and the statistic is not always obvious device [4]. That is the framework in which this study is part which consists of a double measure of natural radioactivity present in soil from two campuses of the University of Douala. A first step of this study was made in Cameroon with a Broad Energy Germanium detector (BEGE-6530 model) and a second with an HPGe (GC0818-7600SL model) detector took place at the Laboratory of Nuclear Physics of the University of Liege in Belgium.

This process is high customizable and there are multiple methods of measuring natural radioactivity based on detection of gamma-rays. This project compared two different types of gamma-ray spectrometers in several different regards. Two different types of germanium detectors are used for two comparative techniques for recording the response of gamma rays exciting electrons. A comparison of the values of the two measures was made in this work in perspective of promoting research and to improve the gamma spectrometry apparatus used in our laboratories.

### Materials and methods

#### Material used

• Two High purity germanium detectors (HPGe) including

- Broad energy germanium detector (including germanium crystal and all protection material)
- High voltage supply
- Analog-to-digital converter (ADC)
- Pre-amplifiers
- Amplifier
- Nuclear Instrumentation Material (NIM)
- Multichannel Analyzer (MCA)

• Sample in cylindrical barker (Including all materials used during sampling campaign, laboratory transfer and sample preparation)
• Global positioning system (GPS used to mark site during sampling campaign)
• Nitrogen cooling system
• Computer including Genie 2000 software and LabSocs mathematics simulation software for calibration
• Calibration sources

#### Sampling and sample preparation

The field experiment was carried out at the two campuses of the University of Douala-Cameroon (04°44′00.1”–04°44′29.7” N and 09°44′00.1”–09°44′45.2” W). Composites of eighteen soil samples were randomly chosen from the two campuses of the University of Douala (seven from Campus1 ESSEC situated at Angel-Raphael and eleven from large area coverage of Campus 2 located at Ndong-Bong Douala-Bassa).

**Fig. 1:**
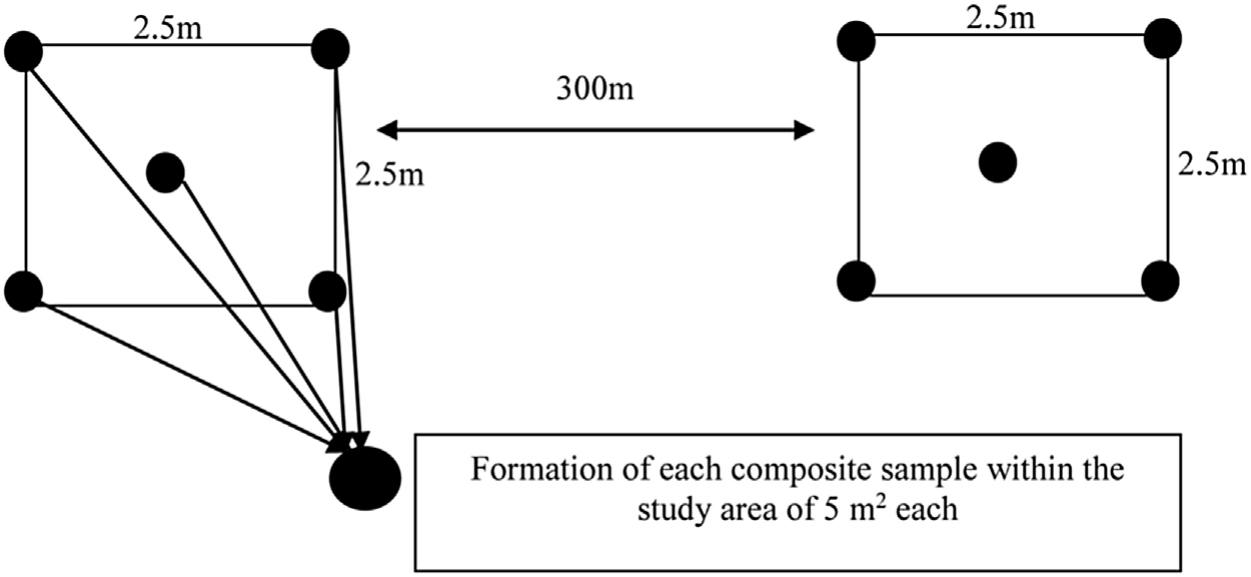
Composite Sample Collection within the Sampling Sites.

The vertical or near vertical surface was dressed to remove smeared soil. This was necessary to minimize the effects of contaminant migration interferences due to smearing of material from other levels. Each composite sample was a mixture of five samples collected within an area of 5 m^2^ separated from each other by a distance of 300 m to cover the study site and to observe a significant local spatial variation in terrestrial radioactivity (see Fig. 1). Each sampling point was marked using a global positioning system (GPS). Four samples were collected at the edges and one at the center. These five samples collected at a depth of approximately 20 cm from the top surface layer were mixed thoroughly to form a composite sample and packed into a polyethylene bag to. At the laboratory, the samples were air-dried for a week then oven-dried at 105 °C for 24 h. The dried samples were grinded into powder and sieved through a 2 mm wire mesh to obtain a uniform particles size. In order to maintain radioactive equilibrium between ^226^Ra and its daughters, the soil samples were then packed in a 120 mL air tight polyethylene cylindrical container, dry-weighed, and stored for a period of 32 days for equilibrium between the long-lived parent and daughter nuclides (For more details, see Ndontchueng et al. [5]). The specifications of the two high purity germanium (HPGe) detectors used are displayed as a part of Table 1.

### Detector calibration procedure: energy and efficiency calibration

The two analyzes use fairly similar methods for calibration of the detectors, but here we mentioned accuracy for each laboratory technics.

*In Douala*, each sample was subjected to a coaxial gamma-ray spectrometer consisting of broad energy germanium detector (BEGe-6530) manufactured by Canberra Industries. Excellent performance, routinely available in coaxial germanium detectors, may be represented by energy resolutions (FWHM) of approximately 0.5 keV at 5.9 keV for ^55^Fe, 2.2 keV at 1332 keV (^60^Co) and approximately 0.75 keV at 122 keV (^57^Co) for the BEGe detector. For these higher efficiency detectors, “peak-to-Compton ratios” are usually quoted in the range of 25 to 40. These ratios are strong functions of resolution, efficiency, and exact detector crystal geometry, and no typical values can be given without knowledge of all of these parameters. The detector is placed in a low-level Canberra Model 747 lead shield with thickness of 10 cm [6]. The energy distributions of the radioactive samples were generated by the computer inbuilt Multiport II Multichannel Analyzer (MCA). Each sample was counted for 86400 s (24 h) for effective peak area statistics of above 0.1%. Following the sample analysis process, the specific activity concentration in Becquerel per kilogram (Bq kg^−1^) for each radionuclide was calculated after background separation using the Genie-2000 software version v3.2 incorporated with cascade summing correction coefficient.

The procedure for extracting Full-Energy Peak Area from the spectral data will be determined by the complexity of the gamma ray spectra as well as the intensity and complexity of the gamma-ray background at energies near the peaks of interest. Assuming a state of secular equilibrium between ^238^U and ^232^Th and their respective decay daughter products, the following relatively intense gamma-ray transitions were used to measure the activity concentrations for the above mentioned radionuclides [5], [7].

(a) ^226^Ra concentration was calculated based on the assumption that it is a weighted mean of the activity concentrations of the gamma-rays of ^214^Pb (295.1 keV, 351.9 keV), ^214^Bi (609.3 keV and 1120.29 keV), and its specific gamma-ray at 186.2 keV. Interference correction due to the presence of 185.7 keV energy peak of ^235^U has been taken into account and subtracted accordingly.
(b) The gamma-ray photopeaks used for the determination of the ^232^Th activity concentration contents were 338.4 keV, 911.2 keV, and 969.11 keV of ^228^Ac and 238.6 keV of ^212^Pb.
(c) ^40^K was directly determined by using its gamma-ray at 1460.8 (10.7%) gamma-ray.

*In Liege*, Each sample was measured with a gamma-ray spectrometer consisting of a high purity germanium detector setup (GC0818-7600SL model) and multichannel analyzer 8192 channel. The system was consisted of a Canberra germanium detector which was shielded to reduce background with active diameter of 43 mm, relative efficiency of 30% at 1.33 MeV ^60^Co line and a resolution of 1.88 keV at the same line. The selected samples were subjected to gamma spectral analysis with a counting time of 86,400 s (24 h). The absolute photopeak efficiency calibration of the system was carried out using standard multi-gamma emitter ^152^Eu source. The sources were placed surrounding the germanium detector with the radionuclides dispersed in gel matrices within planar beakers of geometries identical to that of the evaluated samples. The calibration spectra were also acquired for 7200 s (2 h) [5], [8], [9].

In order to determine the background distribution due to naturally occurring radionuclides in the environment around the detector, an empty polystyrene container was counted in the same manner as the samples prepared in our laboratories. After measurement and subtraction of the background, the activity concentrations were calculated in unit of Bq kg^−1^.

### Measurements of activity concentration

Each sample was counted for a 24 h time and spectra were analysed using Genie 2000 software provides by Canberra Version V.3.2 (BEGe-6530) and version V.3.1 (GC0818-7600SL), including peak search, nuclide identification, activity and uncertainty calculation, and MDA calculation modules software based on the following equation [10]:

(1) $$A(Bq/kg)=\frac{\frac{N_{S}}{t_{S}}-\frac{N_{B}}{t_{B}}}{M_{S}\times \varepsilon \times P_{\gamma }\times K_{SC}\times K_{SA}\times K_{DC}}$$

Where *A*(*Bq*/*kg*)is the activity concentration of radionuclide, $\frac{N_{S}}{t_{S}}$ the count rate of radionuclide in the sample, $\frac{N_{B}}{t_{B}}$ the count rate of radionuclide in the background, *M*_*S*_ the mass of the sample, *ε* the full energy peak efficiency, *P*_*γ*_ the emission probability, *K*_*SC*_ the cascade summing correction, *K*_*SA*_ the correction factor for self-attenuation, K_DC_ the decay correction factor for radionuclide. The uncertainty of the activity concentration (ΔA) was calculated using the following equation:

(2) $$\frac{\Delta A}{A}=\sqrt{{\left(\frac{\Delta N}{N}\right)}^{2}+{\left(\frac{\Delta P_{\gamma }}{P_{\gamma }}\right)}^{2}+{\left(\frac{\Delta \varepsilon }{\varepsilon }\right)}^{2}+{\left(\frac{\Delta M}{¨M}\right)}^{2}}$$

Where ΔN is the count rate uncertainty, ΔPγ the emission probability uncertainty found in the nuclear data tables, Δε the efficiency uncertainty and ΔM the weighing uncertainty [10], [11].

The Minimum Detectable Activity (MDA) calculations based on the following equation:

(3) $$MDA=\frac{(2.71+4.65\times \sqrt{B})\times Decay}{\varepsilon \times b\times LT\times k\times q}$$

Where B = Background sum, Decay = decay factor, ε = efficiency, b = abundance, LT = elapse live time, k = 3700 dps/μCi and q = sample quantity.

Several transitions from decays of shorter-lived radionuclides in the ^238^U decay chain, such as ^214^Pb and ^214^Bi, were also used to estimate the activity concentration of ^226^Ra. The activity concentration of ^232^Th was determined using gamma-ray transitions associated with the decay of ^228^Ac, ^212^Pb and ^208^Tl. Background contributions were also subtracted from the peak areas for the measured samples [12], [13].

The radium-equivalent activity was considered in this case to assess the representative relative uncertainty for specific radioactivity. The radium-equivalent activity is a weighted sum of activities of the ^226^Ra, ^232^Th and ^40^K radionuclides based on the assumption that “370 Bq kg^−1^ of ^226^Ra, 259 Bq kg^−1^ of ^232^Th and 4810 Bq kg^−1^ of ^40^K produce the same gamma-ray dose rate” [5], [14]. It can be calculated by the following relation:

(4) *R_eq_* = *A_Ra_* + 1.43*A_Th_* + 0.077*A_K_*

Where A_Ra_, A_Th_ and A_K_ are the activity concentration of ^226^Ra, ^232^Th and ^40^K in Bq kg^−1^, respectively.

## Method validation

### Radioactivity measurement validation

The activity concentrations of ^226^Ra, ^232^Th and ^40^K in soil samples from the two campuses of the University of Douala-Cameroon have been measured with both spectrometry instruments and presented below in Table 2 with the geological coordinates of each sampling point.

As shown in Table 2, Table 3, the measurement results of the specific activity with the BEGe-6530 detector are very interesting. Indeed, the relative uncertainty is very small compared to the results obtained with the detector GC0818-7600SL regarding ^226^Ra and ^232^Th. It is important to notice that the Broad Energy Germanium Detector is very adaptable to low energies. The gamma ray of ^226^Ra at 186.2 keV is detected with best resolution and minimal uncertainty according to BEGe. However, for potassium which emits a line around 1461 keV, the report Err (7600SL)/Err (BEGE-6530) <1 is less than one. Therefore, the HPGe detector GC0818-7600SL model is more suitable for measuring high gamma energies and not be able at low energies. It can therefore be seen that BEGe measurement results are suitable measures and that we can really use 7600SL only to measure high energies gamma emitters. This is already checked through the computation of the minimum detection activity MDA.

Great information is characterized as being spectral data in which the peaks of interest are well shaped, all around molded and have good “signal to noise." This is a key thought; simply having more data doesn’t enhance the data.

One measure of the quality of a spectrum is the minimum detectable activity (MDA) of the detector system [15], [16]. The resolution, background and efficiency of the detector are related to the MDA. This relationship might be essentially expressed as:

(5) $$MDA(E)\propto \frac{\sqrt{R(E).N(E)}}{\varepsilon (E)}$$

The MDA shifts with energy because the quantities on which it depends change with energy. All the factors in the MDA that exclusively rely on the detector itself were isolated out. For example, the gamma rays per decay, the shield and count time affect the MDA, but will do so in the same way for all detectors. R(E) is the energy resolution of the detector as a function of energy; N(E) is the background counts per keV (unit energy) as a function of energy and ε(E) is the absolute efficiency of the detector and depend on gamma energy. This straightforward is highly huge in directing us towards the right choice of detector using in gamma-ray spectrometry.

In order to reliably measure the gamma-rays emitted from environmental samples (water, air, rock and soil in this case), it is important to achieve as low uncertainties as possible by getting appropriate photon counts. Relative counting uncertainty is defined as reciprocal of the square root of the number of counts (1/(n)^1/2^, n = number of counts), and subsequently, can be diminished by increasing the photon counts. In spite of the acceptable degree of relative uncertainty relies upon investigations, usually under 3.2% of relative uncertainty is viewed just like the minimum value to ensure the unwavering quality of the measurements. The number of photon counts is influenced by measurement time, amount of sample, geometry of samples and detector types.

As shown in Table 3, the relative uncertainty of specific activities for each detector and the ratio of these values for the two detectors shows a relatively larger error for the GC0818-7600SL for ^226^Ra and ^232^Th. These reports ratio between the two detectors ranged from 3.50 to 8.26 with a mean of 5.71 for ^226^Ra and from 1.08 to 1.48 with a mean of 1.32 for ^232^Th, which shows the interest to use the BEGe for these radioisotopes. This ratio is less than one except the case of one sample (UD14), which proves that the use of GC0818-7600SL is more suitable in high energy measurement.

### Validation of the radium equivalent calculation

Table 4 presents the values of radium equivalent activity and uncertainty about the different values. We can see in the last column the uncertainties relative ratio to both facilities calculated by the following equation:

(6) T7600SLBEGe=(ErrReq)7600Sl(ErrReq)BEGe

**Fig. 2:**
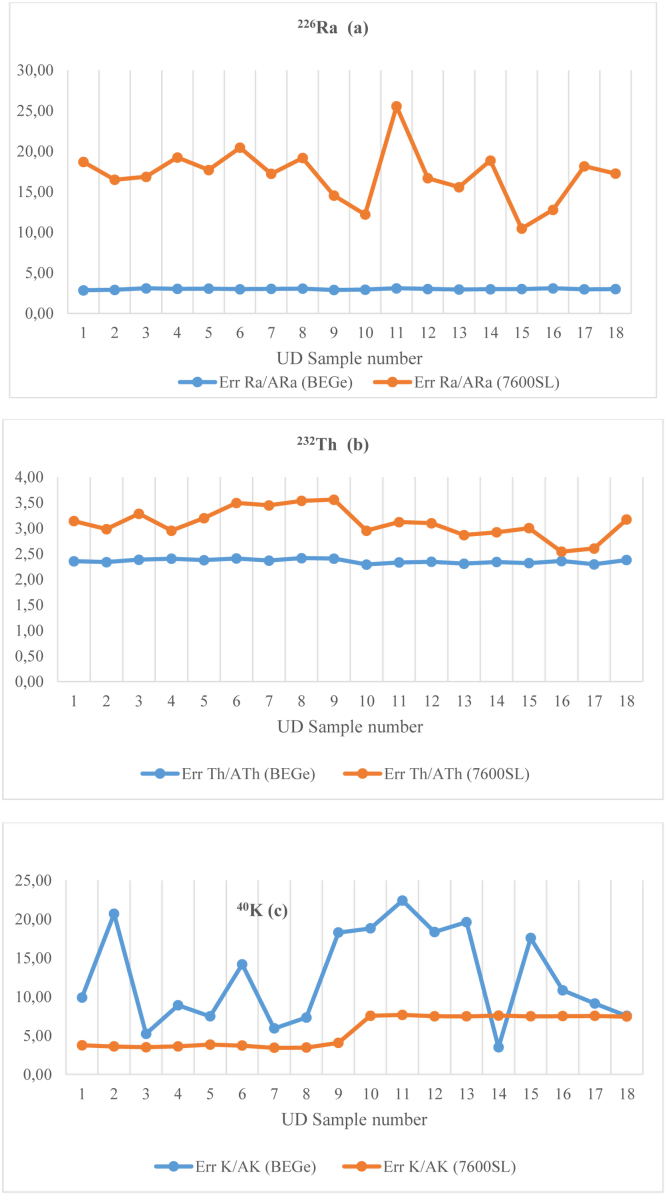
Standard deviation between the two measurements using BEGe-6530 and GC0818-7600SL HPGe detectors: (a) deviation for ^226^Ra, (b) deviation for ^232^Th and (c) deviation for ^40^K.

The average ratio ranged from 2.49 to 4.54 with an average of 3.36; which means that the measurements made with the BEGe are generally more relevant. This variation is also seen in Fig. 2: for 18 samples, uncertainties are higher for GC0818-7600SL.

**Fig. 3:**
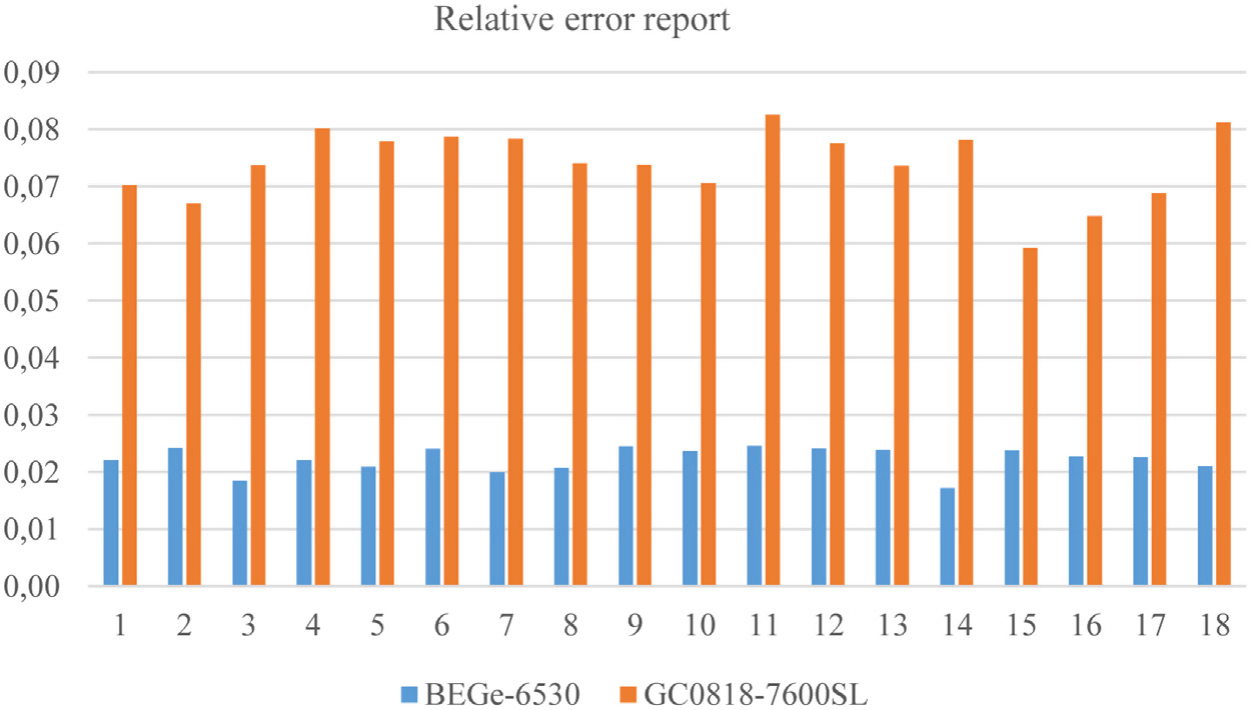
Relative error related to the equivalent radium between the two measures.

Fig. 2 presents a comparison of uncertainties relating to the two types of detector and for different radioisotopes. The fluctuation is almost imperceptible to the measurement of the specific activity of ^226^Ra and ^232^Th with BEGe-6530. But for potassium, the gamma ray is emitted to 1461 Kev, stability is observed rather for GC0818-7600SL. This stability is reflected rather the extent GC0818-7600 as regards the values of ^40^K. A very stable uncertainty is observed for BEGE to the first two curves. Once again, the explanation comes of high and low energies. This is generally reflected in Fig. 3, wherein comparing the standard deviation of radium equivalent activity Ra_eq_ for both types of detectors. It is clear that for measuring natural low level radioactivity the BEGe is more suitable for gamma spectrometry.

The right detector, in this case, is the detector that delivers the most analyzable information in the shortest time for the best statistic measurement. Most spectrometry issues can be tackled with simple detectors. There is no need to have exotic, fascinating or excessively complex designs [15], [16], [17].

## Additional informations

### Poisson statistics applies

For spectrometers that measure and count individual events, such as gamma-rays, counting statistics normally controls the accuracy for measuring the number of events [18], [19]. In the case of a small peak superimposed on a high background in the acquired spectrum, the fluctuation in the background counts degrades the precision with which the net peak counts can be measured. At least, it is this uncertainty in the background counts that determines as far as possible the detection limit for the peak.

It is important to analyze the contribution of counting statistics to the uncertainty in determining the net peak area, and in controlling detection limits. This situation is sometime used in matter-rays Physics like gamma-ray spectrometry. The methodology is applicable to spectrometers that count single events The outcomes proved that it is essential to maximize the peak-to background ratio, the event counting rate, and the counting time to enhance our detection performances and have more reliable results [20]. The latter two parameters enhance accuracy by increasing the number of measured counts.

The above applications normally meet the conditions that characterized the Poisson distribution:

1) The events are uniformly and randomly distributed over the sampling intervals. This is the real principle of Monte Carlo methods used in gamma-ray interaction.

2) The probability of detecting an event during an infinitesimal time interval dt is ρdt, where ρ is the expected counting rate.

3) *pdt* *<<* *1*

4) The probability of detecting more than one event during the infinitesimal time interval dt is negligible.

On the off chance that the events are counted over a finite time period, t, the Poisson Distribution, P(N), describes the probability of recording N counts in a single measurement of duration, t.

(7) $$P(N)=\frac{\mu^{N}e^{-\mu }}{N!}$$

If the measurement is repeated a large number of times, the average value of N approaches the mean of the distribution, μ, as the number of repeated measurements approaches infinity. The Poisson distribution has a standard deviation expressed by the following equation:

(8) $$\sigma_{N}=\sqrt{\mu }\approx \sqrt{N}$$

Substituting N for μ in Eq. (7) recognizes that the value of N from a single measurement is an adequately accurate estimate of μ [21].

A more useful description of the accuracy of the estimation is gotten by multiplying the relative standard deviation, *σ*_*N*_/N, by 100% to express it as

(9) $$\sigma_{N}\%=\frac{\sigma_{N}}{N}\times 100\%=\frac{100\%}{\sqrt{N}}$$

Table 5 shows indicated how the percent standard deviation enhances as the counted number of events increases. Clearly, countless must be accumulated to achieve a precision better than 1%.

Strictly speaking, Eqs. (7) through (9) precisely describe the statistical distribution of events counted only if:

a) dead time losses are negligible, or

b) a perfect lifetime clock is employed to make up for dead time losses [22], [23], [24]. For the ultimate objective of these studies, no less than one of these conditions will be presumed to be fulfilled to our gamma spectrometry framework.

Clearly, it is important to amplify the peak-to-background ratio, the net counting rate in the peak, and the counting time to achieve the lowest detection limits. This same strategy is likewise important for achieving the best relative standard deviation for concentrations well above the detection limit.

### Output of this research

Unmistakably, a few decisions are better than others with regards to picking a detector to quantify natural radioactivity in sand samples with particular energy gamma rays, in a particular geometry and count rate regime. The choice of the best HPGe detector for specific radioactivity estimation circumstance depends on a few basic guidelines. To acquire reliable measurements of radionuclide activity, the knowledge of the detector absolute peak efficiency in the counting conditions is very important.

Background can be lessened by equipping the 10 cm lead shield to obstruct the gamma-ray from outer environment and by applying ultra-low background cryostat materials with low-radioactivity (this case is applied to the BEGe detector of National radiation protection agency of Cameroon).

Broad Energy Germanium detector has a low-form cylindrical shape that is of large detection area, entrance window being made of composite carbon epoxy. Low-form shape has larger solid angle than those of other coaxial type detector (GC0818-7600SL). Subsequently, efficiency in the low energy range is higher than different sorts of detector with comparable relative efficiencies. Theoretically, however, the efficiencies in the high energy range are lower than other coaxial type of similar relative efficiencies. In spite of the fact that, the useful energy range of BEGe detector is from 3 keV to 3 MeV which is smaller than that kind of coaxial HPGe detector (50 keV to 10 MeV), this narrow detection range does not make big difference in measuring the natural radioactivity of environmental samples, the energy ranges of which are mostly below 2 MeV. The acquired results demonstrate that broad energy germanium detector (BEGe-6530 model in this study) is more precise. This conclusion is defended for the accompanying reasons:

- Flat, non- bullettized gems offer ideal efficiencies for samples counted close to the BEGe detector
- Thin, stable entrance window permits the detector to be stored warm with no fear of low energy efficiency loss over time
- The BEGE detector dimensions are for all intents and purposes the same on a model by model basis. This suggests like units can be substituted in an application without complete recalibration and that computer modeling can be done once for each detector size and used for all detectors of that model.
- With cross-sectional areas of 20–65 cm^2^ and thickness of 20–30 mm, the nominal relative efficiency is given underneath close by with the details for whole scope of models. BEGE detectors are ordinarily outfitted with our composite carbon windows which are healthy and provide excellent transmission to underneath 10 keV. Beryllium or aluminum windows are additionally accessible because the transmissive window is formed in the assembly by removing the transition layer material from the central region. Aluminum is more suitable used when there is no interest in energies below 30 keV and enhance toughness is coveted. Beryllium ought to be chosen to take full preferred standpoint of the low energy capability (down to 3 keV) of the BEGE detector [22], [23], [24], [25].

## Figures and Tables

**Table 1:** Specifications of HPGe detector at the National Radiation Protection Agency Laboratory (BEGE-6530) and GC0818-7600SL at the laboratory of nuclear physics at the University of Liege.

Descriptions	Detector	
Detector type (Canberra)	GC0818-7600SL	BEGe-6530
Detector geometry	Plan (coaxial one open end, closed and facing window)	Plan
Detector active area-facing window (mm^2^)	/	6500
Active diameter (mm)	43	91.5
Thickness (mm)	32	31.5
Distance from window (outside) (mm)	5	5
Window thickness (mm)	/	0.6
Detector end-cup type	/	Carbon epoxy
Relative efficiency at 1332.5 of ^60^Co (%)	30	60
Full Width Half Maximum (FWHM) Resolution (keV) at 5.9 KeV	/	0.478
Full Width Half Maximum (FWHM) Resolution (keV) at 122 KeV	0.825	0.695
Full Width Half Maximum (FWHM) Resolution (keV) at 1332.5 KeV	1.88	1.785
Peak/Compton	38	/
Cryostat description	Horizontal dipstick	Vertical dipstick
Peak shape (FWTM/FWHM) for ^60^Co	1.71	1.88

**Table 2:** Specific activities of ^226^Ra, ^232^Th and ^40^K in soil samples from Campus 1 and 2 of the University of Douala measured using BEGe-6530 (Douala) and GC0818-7600SL (Liege) high purity germanium detectors.

Sampling Sites	Sample ID	Latitude	Longitude	Specific activity (Bq kg^−1^)					
				^226^Ra		^232^Th		^40^K	
Laboratory of measurement				Dla^a^ (BEGe)	Lge^b^ (7600SL)	Dla (BEGe)	Lge (7600SL)	Dla (BEGe)	Lge (7600SL)
Campus 1	UD01	04°03′20.8”N	09°43′57.6”W	26.70 ± 0.76	11.20 ± 1.86	65.88 ± 1.55	35.71 ± 0.51	32.56 ± 3.22	117.94 ± 3.80
	UD02	04°03′25.1”N	09°44′00.1”W	28.95 ± 0.84	31.51 ± 2.18	80.03 ± 1.87	54.94 ± 0.57	13.93 ± 2.88	195.72 ± 4.07
	UD03	04°03′22.6”N	09°44′07.1”W	21.99 ± 0.68	28.89 ± 2.18	59.14 ± 1.41	28.83 ± 0.49	70.89 ± 3.70	218.30 ± 4.13
	UD04	04°03′19.7”N	09°44′04.1”W	25.44 ± 0.77	28.38 ± 2.45	63.27 ± 1.52	27.37 ± 0.43	38.01 ± 3.38	170.06 ± 3.94
	UD05	04°03′17.2”N	09°44′02.9”W	23.27 ± 0.71	29.04 ± 2.33	59.78 ± 1.42	29.94 ± 0.49	44.03 ± 3.29	93.82 ± 3.74
	UD06	04°03′14.8”N	09°44′08.0”W	29.17 ± 0.87	21.52 ± 2.33	71.06 ± 1.71	45.44 ± 0.60	21.82 ± 3.09	187.97 ± 4.10
	UD07	04°03′16.7”N	09°44′11.0”W	22.82 ± 0.69	39.59 ± 2.55	62.57 ± 1.48	31.28 ± 0.53	52.80 ± 3.12	254.53 ± 4.27
Minimum				21.99 ± 0.68	11.20 ± 1.86	59.14 ± 1.41	27.37 ± 0.43	13.93 ± 2.88	93.82 ± 3.74
Maximum				29.17 ± 0.87	39.59 ± 2.55	65.88 ± 1.55	54.94 ± 0.57	70.89 ± 3.70	254.53 ± 4.27
Average values ± Standard Deviation				25.48 ± 0.92	27.16 ± 2.27	65.96 ± 7.39	36.22 ± 0.52	39.15 ± 19.14	176.91 ± 4.01
Campus 2	UD08	04°03′29.7”N	09°44′26.5”W	22.27 ± 0.68	13.89 ± 2.00	52.60 ± 1.27	30.17 ± 0.53	44.70 ± 3.27	248.63 ± 4.28
	UD09	04°03′31.0”N	09°44′30.3”W	27.68 ± 0.80	41.61 ± 2.18	62.79 ± 1.51	32.50 ± 0.54	16.76 ± 3.06	47.38 ± 3.60
	UD10	04°03′22.0”N	09°44′30.0”W	24.94 ± 0.73	92.88 ± 3.06	72.50 ± 1.66	69.02 ± 0.55	14.68 ± 2.76	225.98 ± 4.65
	UD11	04°03′25.1”N	09°44′36.8”W	21.99 ± 0.68	11.79 ± 2.55	63.93 ± 1.49	76.01 ± 0.67	11.89 ± 2.66	172.56 ± 4.21
	UD12	04°03′21.5”N	09°44′39.0”W	22.89 ± 0.69	50.66 ± 2.87	64.46 ± 1.51	69.23 ± 0.71	15.82 ± 2.90	238.96 ± 4.63
	UD13	04°03′16.5”N	09°44′39.8”W	25.87 ± 0.76	69.77 ± 3.17	74.12 ± 1.71	91.41 ± 0.70	15.10 ± 2.96	225.65 ± 4.52
	UD14	04°03′18.4”N	09°44′37.5”W	23.84 ± 0.71	41.51 ± 2.91	63.27 ± 1.48	78.10 ± 0.75	80.76 ± 2.80	198.16 ± 4.41
	UD15	04°03′16.8”N	09°44′35.5”W	26.74 ± 0.80	49.89 ± 1.75	78.99 ± 1.83	66.69 ± 0.65	18.29 ± 3.21	260.74 ± 4.78
	UD16	04°03′24.9”N	09°44′42.2”W	24.64 ± 0.76	62.85 ± 2.46	71.66 ± 1.69	73.85 ± 0.59	29.94 ± 3.24	244.97 ± 4.59
	UD17	04°03′21.2”N	09°44′45.2”W	24.98 ± 0.74	23.45 ± 2.18	72.39 ± 1.66	76.32 ± 0.60	19.84 ± 1.81	240.18 ± 4.56
	DU18	04°03′18.2”N	09°44′42.7”W	23.67 ± 0.71	49.43 ± 288	57.20 ± 1.36	59.77 ± 0.65	42.26 ± 3.18	271.76 ± 4.74
Minimum				21.99 ± 0.68	11.79 ± 2.55	52.60 ± 1.27	30.17 ± 0.53	11.89 ± 2.66	47.38 ± 3.60
Maximum				27.68 ± 0.80	92.88 ± 3.06	78.99 ± 1.83	91.41 ± 0.70	80.76 ± 2.80	271.76 ± 4.74
Average values ± Standard Deviation				24.50 ± 1.80	46.16 ± 2.55	66.72 ± 7.91	65.73 ± 0.65	28.19 ± 20.72	215.91 ± 4.45
Worldwide	Range			17.00−60.00		11.00−68.00		140.00−850.00	
	Average			35.00		30.00		400.00	

a Dla means Measured at the laboratory of the University of Douala.

b Lge means measured at the laboratory of the University of Liege.

**Table 3:** Errors related to Specific activities of ^226^Ra, ^232^Th and ^40^K and standard deviation in soil samples from Campus 1 and 2 using both detectors.

sample Id	Err Ra/Ara (BEGe)	Err Ra/Ara (7600SL)	Ra7600SL/BEGe	Err Th/Ath (BEGe)	Err Th/Ath (7600SL)	Th7600SL/BEGe	Err K/AK (BEGe)	Err K/AK (7600SL)	K7600SL/BEGe
UD1	2.85	18.67	6.56	2.35	3.14	1.33	9.89	3.74	0.38
UD2	2.90	16.48	5.68	2.34	2.98	1.28	20.67	3.59	0.17
UD3	3.09	16.85	5.45	2.38	3.28	1.38	5.22	3.49	0.67
UD4	3.03	19.23	6.35	2.40	2.95	1.23	8.89	3.60	0.41
UD5	3.05	17.69	5.80	2.38	3.20	1.35	7.47	3.83	0.51
UD6	2.98	20.44	6.85	2.41	3.49	1.45	14.16	3.70	0.26
UD7	3.02	17.22	5.69	2.37	3.45	1.46	5.91	3.42	0.58
UD8	3.05	19.18	6.28	2.41	3.53	1.46	7.32	3.44	0.47
UD9	2.89	14.54	5.03	2.40	3.56	1.48	18.26	4.05	0.22
UD10	2.93	12.20	4.17	2.29	2.95	1.29	18.80	7.53	0.40
UD11	3.09	25.55	8.26	2.33	3.12	1.34	22.37	7.65	0.34
UD12	3.01	16.67	5.53	2.34	3.10	1.32	18.33	7.48	0.41
UD13	2.94	15.58	5.30	2.31	2.86	1.24	19.60	7.47	0.38
UD14	2.98	18.86	6.33	2.34	2.92	1.25	3.47	7.56	2.18
UD15	2.99	10.46	3.50	2.32	3.00	1.29	17.55	7.47	0.43
UD16	3.08	12.78	4.14	2.36	2.54	1.08	10.82	7.49	0.69
UD17	2.96	18.15	6.13	2.29	2.60	1.14	9.12	7.53	0.83
UD18	3.00	17.25	5.75	2.38	3.17	1.33	7.52	7.44	0.99
Min	2.85	10.46	3.50	2.29	2.54	1.08	3.47	3.42	0.17
Max	3.09	25.55	8.26	2.41	3.56	1.48	22.37	7.65	2.18
Average	2.99	17.10	5.71	2.36	3.10	1.32	12.52	5.58	0.57

**Table 4:** Errors related to equivalent radium of ^226^Ra, ^232^Th and ^40^K and standard deviation in soil samples from Campus 1 and 2 using both detectors.

Sample Id	R_eq_ (Bq kg^−1^)		Err R_eq_		err/R_eq_		T_7600SL/BEGE_
	BEGe-6530	7600SL	BEGe-6530	7600SL	BEGe-6530	7600SL	
UD1	145.98	41.02	3.22	2.88	0.02	0.07	3.18
UD2	154.12	49.31	3.74	3.31	0.02	0.07	2.77
UD3	161.15	43.40	2.98	3.20	0.02	0.07	3.98
UD4	145.18	42.00	3.20	3.37	0.02	0.08	3.63
UD5	142.66	42.61	2.99	3.32	0.02	0.08	3.71
UD6	147.59	44.49	3.55	3.50	0.02	0.08	3.27
UD7	152.95	46.41	3.05	3.64	0.02	0.08	3.93
UD8	131.91	41.87	2.75	3.10	0.02	0.07	3.56
UD9	130.37	43.95	3.19	3.24	0.02	0.07	3.01
UD10	139.92	66.99	3.32	4.73	0.02	0.07	2.98
UD11	122.57	51.01	3.02	4.21	0.02	0.08	3.36
UD12	127.25	59.12	3.07	4.59	0.02	0.08	3.21
UD13	143.49	67.10	3.43	4.94	0.02	0.07	3.08
UD14	176.50	58.69	3.04	4.59	0.02	0.08	4.54
UD15	153.78	57.47	3.66	3.41	0.02	0.06	2.49
UD16	150.17	62.07	3.43	4.02	0.02	0.06	2.84
UD17	143.77	54.37	3.25	3.75	0.02	0.07	3.04
UD18	138.01	55.96	2.90	4.55	0.02	0.08	3.87
Min	122.57	41.02	2.75	2.88	0.02	0.06	2.49
Max	176.50	67.10	3.74	4.94	0.02	0.08	4.54
Average	144.85	51.55	3.21	3.80	0.02	0.07	3.36

**Table 5:** *σ*_*N*_% for selected values of N.

N	*σ*_*N*_%
1	100.0%
100	10.0%
10,000	1.0%
1,000,000	0.1%
100,000,000	0.01%
10,000,000,000	0.001%
